# Underuse of recommended treatments among people living with treatment-resistant psychosis

**DOI:** 10.3389/fpsyt.2022.987468

**Published:** 2022-09-06

**Authors:** Julia M. Lappin, Kimberley Davies, Maryanne O'Donnell, Ishan C. Walpola

**Affiliations:** ^1^The Tertiary Referral Service for Psychosis (TRSP), South Eastern Sydney Local Health District, Randwick, NSW, Australia; ^2^Discipline of Psychiatry and Mental Health, School of Clinical Medicine, UNSW Medicine & Health, UNSW Sydney, Kensington, NSW, Australia

**Keywords:** psychosis, treatment-resistance, clozapine, ECT, schizophrenia

## Abstract

**Background:**

International guidelines recommend that individuals with treatment-resistant psychosis must be treated with clozapine. ECT has also been reported to improve symptom profiles. Identification of clozapine and/or ECT use in real-world practice enables understanding of the extent to which this evidence-base is implemented.

**Setting:**

Statewide public health tertiary referral service, the Tertiary Referral Service for Psychosis (TRSP), NSW, Australia.

**Objectives:**

To (i) describe clinical characteristics of individuals with treatment-resistant psychosis and to detail the proportion who had received a trial of clozapine or ECT at any point during their illness course; (ii) describe the characteristics of the treatment trials in both those currently on clozapine and those previously on clozapine; (iii) document reasons in relevant individuals why clozapine had never been used.

**Methods:**

All TRSP clients who met the criteria for treatment resistance (TR) were included. A detailed casenote review was conducted to examine whether clozapine and/or ECT had ever been prescribed. Characteristics of clozapine and ECT trials were documented. Tertiary service treatment recommendations are described.

**Findings:**

Thirty-six of 48 individuals had TR. They had marked clinical and functional impairment. A minority were currently receiving clozapine (*n* = 14/36). Most had received a clozapine trial at some point (*n* = 32/36). Most experienced persistent clinical symptoms while on clozapine (*n* = 29/32). Clozapine plasma levels were very rarely reported (4/32). Augmentation and antipsychotic polypharmacy were common among those currently on clozapine. The median clozapine trial duration was 4.0 (IQR: 3.0–20.3) months in individuals previously prescribed clozapine. Reasons for clozapine discontinuation included intolerable side effects (*n* = 10/18) and poor adherence (*n* = 7/18). One-quarter of TR individuals had trialed ECT (*n* = 9/36). Tertiary service recommendations included routine plasma monitoring to optimize dose among people currently on clozapine; clozapine retrial in those previously treated; and clozapine initiation for those who had never received clozapine. ECT was recommended to augment clozapine and as an alternative where clozapine trial/retrial was not feasible.

**Conclusion:**

Among people with TR referred to a tertiary service, clozapine and ECT were underutilized. Clozapine trials are typically terminated without an adequate trial. Strategies to optimize the use of clozapine therapy and ECT in clinical settings are needed to increase the therapeutic effectiveness of evidence-based therapies for treatment-resistant psychosis.

## Introduction

Treatment-resistant psychotic illness is one of the greatest therapeutic challenges in psychiatry, affecting at least one-third of individuals who experience psychotic illness (1). Treatment-resistant psychosis or schizophrenia (TRS) has been historically variously defined with resultant heterogeneity in studies investigating outcomes, prompting the development of the TRRIP (Treatment Response and Resistance in Psychosis) consensus definition of TRS as “an inadequate clinical response to sequential treatment with at least two different antipsychotics at an adequate dose, duration, and adherence” (2). Since its demonstration of superior effectiveness decades ago (3), clozapine remains the only drug treatment licensed for the treatment of TRS, endorsed in clinical guidelines internationally (4–7). Benefits associated with clozapine use include reduced mortality (8), reduced symptom profile (9), and reduced frequency of subsequent hospital admissions (10) with associated significant health cost savings (11).

Despite the established evidence-base for clozapine's benefits, it is underutilized, as demonstrated by both low rates of use among populations with TRS (12) and long delays in clozapine initiation following the detection of TRS (13, 14). This is concerning as, in the face of non-response, antipsychotic doses are given at higher doses or there is the use of polypharmacy with resultant increased adverse side effects (13). Studies conducted in real-world settings have enabled a better understanding of clozapine's clinical utility and cost-effectiveness (15, 16). Much remains unknown, however, about the optimization of clozapine trials in real-world settings, for example, whether adequate dosing of clozapine is achieved through, for example, systematically assessing clinical response and regularly monitoring clozapine plasma levels. This is important because if clozapine is not adequately trialed then failure to respond may erroneously be assumed to be clozapine-resistance when in fact it may represent pseudo-resistance (15, 16). In such instances, a therapeutic plasma level over an adequate duration of treatment may have led to clinical improvement and clozapine response. Consensus recommendations are that clozapine levels = >350 ng/ml should be achieved to exclude pseudo-resistance and that an adequate trial of clozapine should be of a minimum of 3 months duration after achieving therapeutic plasma levels (2, 17).

Current evidence suggests that approximately 40% of patients with TRS achieve an adequate response to clozapine in meta-analyses of clinical trials, suggesting that the remainder will be “ultra-resistant” (9). For this group of individuals, a limited evidence base exists to guide ongoing treatment decisions. There is meta-analytic support for the augmentation of clozapine, that is, the addition of an additional pharmacological agent. While earlier meta-analyses did not demonstrate an effect in available double-blind placebo-controlled studies (18), a small effect of augmentation strategies over clozapine alone was evident when trials lasted for longer than 10 weeks (19). Later studies of clozapine augmentation with higher doses of amisulpride or aripiprazole appear to drive a larger, albeit still small (effect size = 0.24), reduction in symptoms (20). Other augmentation agents for which there is some evidence include sodium valproate (21, 22), lamotrigine (23) [though this has been attributed to the influence of two outlying studies (22)], and fluoxetine (21). Risperidone has one RCT supporting its use as an augmentation strategy (24), but two separate RCTs were unable to demonstrate evidence of benefit (25, 26). Guidelines recommend that augmentation agents should be introduced only after ensuring that clozapine levels have been optimized and psychotic symptoms reassessed (17).

Finally, the cytochrome P450 (CYP) 1A2 receptor inhibitor fluvoxamine may be used in some clinical practice as an adjunct to clozapine by increasing plasma blood levels (27). This combination may decrease plasma levels of the metabolite norclozapine while increasing levels of clozapine, and so increase the plasma clozapine:norclozapine ratio that has been implicated in side effects limiting the tolerability of clozapine (28).

Another approach where inadequate benefit has been achieved with clozapine, or if clozapine is not tolerated, is to use electroconvulsive therapy (ECT) for TRS. The meta-analytic-level evidence—limited by the quality of the individual trials included—suggests moderate-quality RCT evidence for a positive effect of ECT on medium-term clinical response in TRS (29). ECT can also be employed as an augmentation agent for clozapine where clozapine resistance is identified, according to a large meta-analysis including studies from a predominantly Chinese database, with notable concerns about individual study methodology and evidence of publication bias at post-ECT assessment, but not at end-point assessment (28).

Better understanding is needed for current real-world practice in managing TRS. The aims of this study, in a population of people with complex and difficult to treat psychotic illness referred to as tertiary service, were to (i) describe clinical characteristics of individuals with treatment-resistant psychosis and to detail the proportion who had received a trial of clozapine or ECT at any point during their illness course, (ii) describe the characteristics of the treatment trials in both those currently on clozapine and those previously on clozapine, (iii) document reasons in relevant individuals why clozapine had never been used. Finally, we report treatment recommendations by the group following their assessment in a tertiary referral service for psychosis, with a focus on pharmacological recommendations.

## Materials and methods

### Setting

The Tertiary Referral Service for Psychosis (TRSP) is a state-wide publicly funded mental health tertiary referral outpatient service in New South Wales, Australia. The TRSP provides specialist consultation to mental health teams supporting people who live with a complex or difficult-to-treat psychotic illness. The TRSP conducts multidisciplinary assessments and provides individualized treatment recommendations for all individuals referred by their public mental health service.

Individuals accepted for assessment must meet the following criteria: 16 years of age or older; has an enduring psychotic illness or psychosis-like experiences that require diagnostic review; not currently in a crisis; currently engaged with a public mental health service. Further, the individual consents to participate in assessments; and the referring service acknowledges responsibility for the ongoing clinical care of the individual. Indications for referral include but are not limited to: long duration of illness; high illness burden in terms of symptoms severity and functional impairment; non-response to appropriate pharmacological or psychosocial treatments; and presence of complicating factors (e.g., comorbid health conditions and intellectual disability). The aim of the service is to support clinicians working with people with complex and difficult to treat psychosis to optimize management, including both pharmacological and non-pharmacological approaches. The TRSP is promoted through clinical networks and local and national fora. More information about the service is provided on the website: http://www.mindgardens.org.au/phase-1-services/mindgardens-clinic.

### Study design

A retrospective casenote review was conducted of all individuals undergoing multidisciplinary team assessments with the TRSP between 22nd June 2020 and 23rd May 2022. Psychiatric assessments were conducted by the team consultant psychiatrist and registrar. Physical health assessments were conducted by the team general practitioner. This project was reviewed and approved as a quality improvement project by South-Eastern Sydney Local Health District (Ref.: T20/81560).

### Measures

The diagnosis was made during consensus clinical meetings according to the International Classification of Diseases (ICD)-10 diagnostic criteria (30), based on clinical interviews and through extensive collateral information-gathering from clinicians, supports, and family members. Psychopathology at the time of the TRSP review was assessed using the Clinical Global Impression Schizophrenia Scale (CGI-SCH) (31). Socio-occupational functioning was assessed using the Social and Occupational Functioning Assessment Scale (SOFAS), Health of Nation Outcome Scales (HoNOS), and Life Skills Profile-16 (LSP-16).

### Data extraction

A retrospective case history examination was conducted of medical records, medication charts, and clinical documentation. Demographic information was collated for all participants, as were key clinical measures including past-year tobacco, alcohol, and other substance use; duration of psychotic illness; start and end dates of prescribed antipsychotic medication, including dosage and adherence to treatment; and history of ECT trials. Treatment resistance (TR) was determined as present if minimum requirement TRRIP criteria were met, that is, inadequate clinical response to sequential treatment with at least two different antipsychotics at an adequate dose, duration, and adherence (2). Specifically, TR was deemed to be present where individuals continued to have persistent psychotic symptoms of at least moderate severity and functional impairment despite having received at least two sequential antipsychotic trials, each of at least 6 weeks duration at a daily dose of 600 mg of chlorpromazine equivalents (2, 32). The onset of TR was recorded categorically as within 2 years of illness onset, or later.

Details of clozapine use, past and present, were extracted including dose, duration of the trial, and therapeutic plasma level. Use of augmentation agents or the adjunctive agent fluvoxamine and polypharmacy were extracted for individuals currently taking clozapine. The following were considered possible augmentation agents: aripiprazole, amisulpride, sodium valproate, lithium, lamotrigine, and fluoxetine. Polypharmacy was defined as the use of one or more antipsychotic treatments in addition to clozapine, other than those detailed as possible augmentation agents. Reasons for non-initiation were recorded for individuals who never commenced clozapine, and reasons for discontinuation for individuals with previous use. For both groups, evidence of clozapine plasma levels being conducted during a clozapine trial was recorded, and whether these reached the response threshold of 350 ng/ml (33, 34).

For all individuals, information related to the use of ECT was recorded, including indications for ECT trial, an average number of courses, and documented evidence of symptom improvement.

Treatment recommendations arising from the TRSP assessment process were recorded in the following categories: psychopharmacological (including clozapine trial/retrial, changes related to clozapine dose, augmentation, and other psychopharmacological treatments, such as mood stabilizer or antidepressant); ECT; psychological (including cognitive behavioral therapy for psychosis, cognitive remediation therapy, other); physical health (including commencement on medications for metabolic syndrome, screening for bloodborne viruses, smoking cessation aids, among other); psychosocial (including inpatient and community supports, e.g., behavioral support practitioner, occupational therapist, and access to rehabilitation care); substance use interventions; support for disability package (the National Disability Insurance Scheme in the Australian setting); and other (including immunological treatment; guardianship and specialist medical referrals). All data were recorded in a secure online database.

### Data analysis

Individuals were assigned to one of three groups: (i) never prescribed clozapine, (ii) previously prescribed clozapine; or (iii) currently prescribed clozapine.

Demographic characteristics, characteristics of treatment-resistance, measures of clozapine use, and treatment recommendations are reported using descriptive and frequency statistics. The Shapiro-Wilk test was used to test continuous data for normality. Means and standard deviations are reported where the data are normally distributed, while medians with interquartile range values are reported for non-parametric data.

## Results

Forty-eight individuals were assessed, of whom 36 (75.0%) were identified to have treatment-resistant (TR) psychosis. Across all three groups, there was a predominance of males with diagnoses almost entirely of schizophrenia or schizoaffective disorder (Table 1). The mean duration of psychotic illness was over 20 years. Where the timing of onset of TR was known, two in three had developed TR within 2 years of first psychosis onset. The group demonstrated moderate global clinical impairment with median positive symptoms in the markedly ill range (CGI of 5.0) and function typically in the major impairment range (mean SOFAS score 35.7). None of the individuals was in any form of employment, and only one-quarter were considered capable of sheltered work (Table 1).

**Table 1:** Demographic and clinical characteristics in treatment-resistant (TR) sample.

	**Total TR**	**Currently**	**Previously**	**Never prescribed**
	**sample**	**prescribed clozapine**	**prescribed clozapine**	**prescribed clozapine**
	**(*n* = 36)**	**(*n* = 14)**	**(*n* = 18)**	**(*n* = 4)**
Age (M, SD)	42.4 (14.5)	37.2 (10.8)	49.5 (14)	28.5 (12.2)
Male Gender (*%, n*)	63.9 (23)	78.6 (11)	50.0 (9)	75.0 (3)
Country of birth Australia (*%, n*)	86.1 (31)	78.6 (11)	94.4 (17)	75.0 (3)
**Diagnosis and clinical characteristics**
Schizophrenia (*%, n*)	61.1 (22)	64.3 (9)	61.1 (11)	50.0 (2)
Schizoaffective disorder (*%, n*)	36.1 (13)	35.7 (5)	33.3 (6)	50.0 (2)
Bipolar disorder (*%, n*)	2.8 (1)	0	5.6 (1)	0
Duration psychotic illness (yrs, M, SD)	22.7 (13.1)	18.2 (10.8)	27.3 (13.6)	15.3 (13.8)
**Onset of TR Known (** ***%, n*** **)**	77.8 (28)	85.7 (12)	66.7 (12)	100 (4)
Within up to 2 years from onset	52.8 (19)	64.3 (9)	50.0 (9)	25.0 (1)
Greater than 2 years from onset	25.0 (9)	21.4 (3)	16.7 (3)	75.0 (3)
**CGI overall severity (Mdn, IQR)**	4.0 (4.0–5.0)	4.5 (4.0–6.0)	5.0 (4.0–5.3)	3.0 (3.0–4.0)
CGI Positive symptoms	5.0 (4.0–6.0)	5.0 (4.0–7.0)	5.0 (5.0–6.0)	3.0 (2.0–4.0)
CGI Negative symptoms	3.0 (2.0–4.0)	3.5 (2.0–4.3)	3.5 (2.8–5.0)	3.0 (2.0–3.8)
CGI Depressive symptoms	3.0 (2.0–4.0)	2.0 (2.0–4.0)	3.0 (2.8–4.0)	3.0 (2.0–4.0)
CGI Cognitive symptoms	4.0 (3.0 −5.0)	4.0 (4.0–5.0)	4.0 (3.0–5.0)	3.0 (2.0–4.0)
**Functioning and co–morbidity**
Physical health comorbidities (*%, n*)	69.4 (25)	71.4 (10)	83.3 (15)	0
Alcohol or other substance use (*%, n*)	25.0 (9)	35.7 (5)	16.7 (3)	25.0 (1)
Tobacco use (*%, n*)	52.8 (19)	71.4 (10)	44.4 (8)	25.0 (1)
SOFAS (M, SD)	35.7 (13.3)	32.3 (15.8)	38.1 (12.5)	36.6 (6.3)
HoNOS (M, SD)	30.3 (6.3)	29.9 (7.1)	29.5 (5.6)	35.0 (5.4)
LSP−16 (M, SD)	30.0 (7.7)	31.1 (9.5)	28.8 (6.4)	31.8 (7.4)
Employment capability (*%, n*)	25.0 (9)	28.6 (4)	27.8 (5)	0

Over two-thirds of the group reported comorbid physical health conditions, including dyslipidemia (25.0%, *n* = 9), type 2 diabetes (25.0%, *n* = 9), epilepsy (11.0%, *n* = 4), obesity (11.0%, *n* = 4), obstructive sleep apnea (11.0%, *n* = 4), and hepatitis C (6.0%, *n* = 2).

### Currently prescribed clozapine

The 14 individuals who were currently prescribed clozapine were predominantly males with a mean age in the late 30's (Table 1). Three-quarters had developed TR within 2 years of psychosis onset. Rates of physical health comorbidities and tobacco smoking were very high, and over one-third had alcohol or other substance misuse (Table 1).

The median duration of the clozapine trial was 48 months (IQR 14.0–126.0) and the median dose was 312.5 mg (IQR 243.8–418.8) (Table 2). Plasma levels were very rarely recorded. Where available, evidence that a therapeutic level ≥350 ng/ml had been reached was present in only one individual (Table 2).

**Table 2:** Clozapine and ECT characteristics in individuals currently and previously prescribed clozapine.

	**Clients currently prescribed**		**Clients previously prescribed**	
	**clozapine (*****n =*** **14)**		**clozapine (*****n =*** **18)**	
**Average dose (mg)**
Mean (SD)	*n =* 14	337.7 (130.7)	*n =* 12	258.3 (160.7)
Median (IQR)	*n =* 14	312.5 (243.8–418.8)	*n =* 12	225.0 (150.0–337.5)
**Duration prescribed clozapine (Months)**
Mean (SD)	*n =* 13	69.1 (64.0)	*n =* 16	28.6 (53.3)
Median (IQR)	*n =* 13	48.0 (14.0–126.0)	*n =* 16	4.0 (3.0–20.3)
**Therapeutic plasma level reached (%)**
Yes	*n =* 1	7.1	*n =* 2	11.1
No	*n =* 1	7.1	*n =* 0	0
Not known	*n =* 12	85.8	*n =* 16	88.9
Symptoms persisted whilst prescribed clozapine (%)	*n =* 13	92.9	*n =* 16	88.9
Received ECT (%)	*n =* 4	28.6	*n =* 5	27.8
Courses of ECT (Mdn, IQR)	*n =* 3	4.0	*n =* 3	2.5

Over half (*n* = 8/14; 57.1%) of individuals currently prescribed clozapine were also prescribed medications that could be used for augmentation, including sodium valproate (21.4%, *n* = 3), combined sodium valproate and fluvoxamine (7.1%, *n* = 1), combined sodium valproate and lithium (7.1%, *n* = 1); fluoxetine (7.1%, *n* = 1); lithium (7.1%, *n* = 1); or lamotrigine (7.1%, *n* = 1). None was prescribed either aripiprazole or amisulpride in addition to clozapine. The adjunctive agent fluvoxamine was prescribed in a further two individuals (14.3%, *n* = 2). Polypharmacy was common: over half (57.1%, *n* = 8) were prescribed at least one other antipsychotic medication, of whom two were prescribed two additional antipsychotics. These included olanzapine (*n* = 3), paliperidone (*n* = 2), flupenthixol (*n* = 2), risperidone (*n* = 1), chlorpromazine (*n* = 1), and quetiapine (*n* = 1).

Four (28.6%) individuals had received ECT at some point in their illness course. Where documented, the indication for ECT was treatment-resistant psychosis (*n* = 3; 100%). Information on response to ECT was available for three (75.0%): in one individual no moderate severity symptoms persisted and in a further two, more than one moderate symptom persisted but with overall improvement.

### Previously prescribed clozapine

Eighteen individuals had previously been prescribed clozapine (Table 1). The mean age was in the late 40s and the mean duration of psychotic illness was 27.3 years. Three-quarters had developed TR within 2 years of psychosis onset. Physical health comorbidities were almost universal (83.3%) with high rates of tobacco smoking (Table 1). The median duration of the clozapine trial was very short at 4 months with a large range across individuals (Table 2). Plasma levels were recorded in only two individuals. Evidence that the therapeutic plasma level reached ≥350 ng/ml was present in *n* = 2 (Table 2).

The two main reasons cited for clozapine discontinuation were adverse effects (55.6%; *n* = 10) and non-adherence/self-cessation (38.9%; *n* = 7). Clozapine was discontinued in one further individual because of limited benefit to symptoms (5.6%). Adverse side effects cited as rationale for discontinuation could be multiple and included myocarditis (30.0%, *n* = 3), neutropenia (20.0%, *n* = 2), agranulocytosis (10.0%, *n* = 1), sialorrhoea (20.0%, *n* = 2), and other breathing and heart related effects (30.0%, *n* = 3), including atrial fibrillation (10.0%, *n* = 1), sinus tachycardia (10.0%, *n* = 1), and nocturnal shortness of breath (10.0%, *n* = 1). Other side effects (50.0%, *n* = 5) included sedation (*n* = 1), client complaints of somatic symptoms (*n* = 1), myoclonic movements and dysarthria (*n* = 1), worsening symptoms of other mental illness (*n* = 1), and pyrexia (*n* = 1).

Five individuals (27.8%) had ever received ECT. Documented indications were treatment-resistant psychosis (*n* = 3; 60.0%) and other (including suicidal ideation) (*n* = 2; 40.0%). In all for whom information on response to ECT was available (*n* = 4), more than one moderate symptom persisted but there was an overall improvement.

### Never prescribed clozapine

Only four individuals with TR had never been prescribed clozapine (Table 1). They were predominantly males in the late 20s, with TR onset occurring later than 2 years post psychosis onset in the majority. They were unusual in the cohort in having no physical health comorbidities (Table 1). Reasons for never having been prescribed clozapine included chaotic lifestyle with polysubstance use and medication non-adherence (*n* = 1); diagnosis of TR psychosis made only at TRSP assessment (*n* = 1); undocumented due to management in the private health sector for many years until a recent transfer to public mental health services (*n* = 2). None had received ECT.

### TRSP treatment recommendations

**Figure 1:**
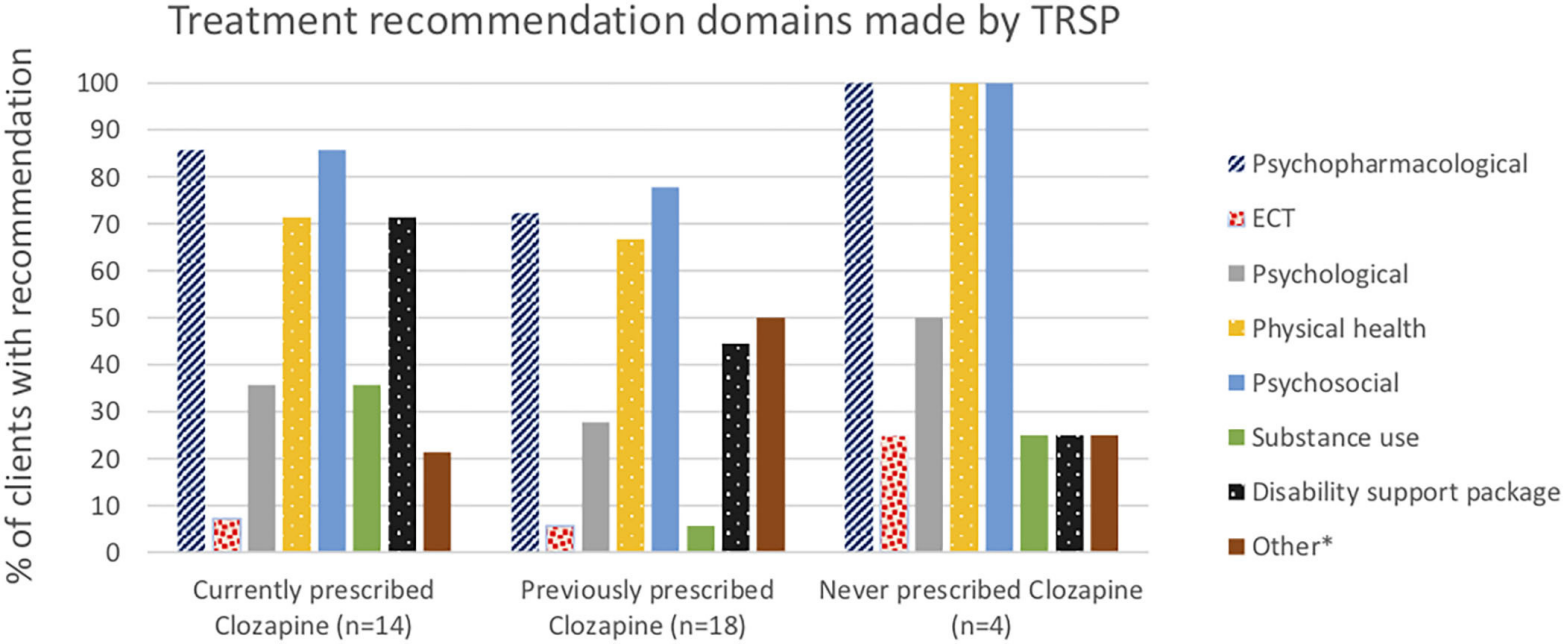
Domains of treatment recommendations made by TRSP.

**Figure 2:**
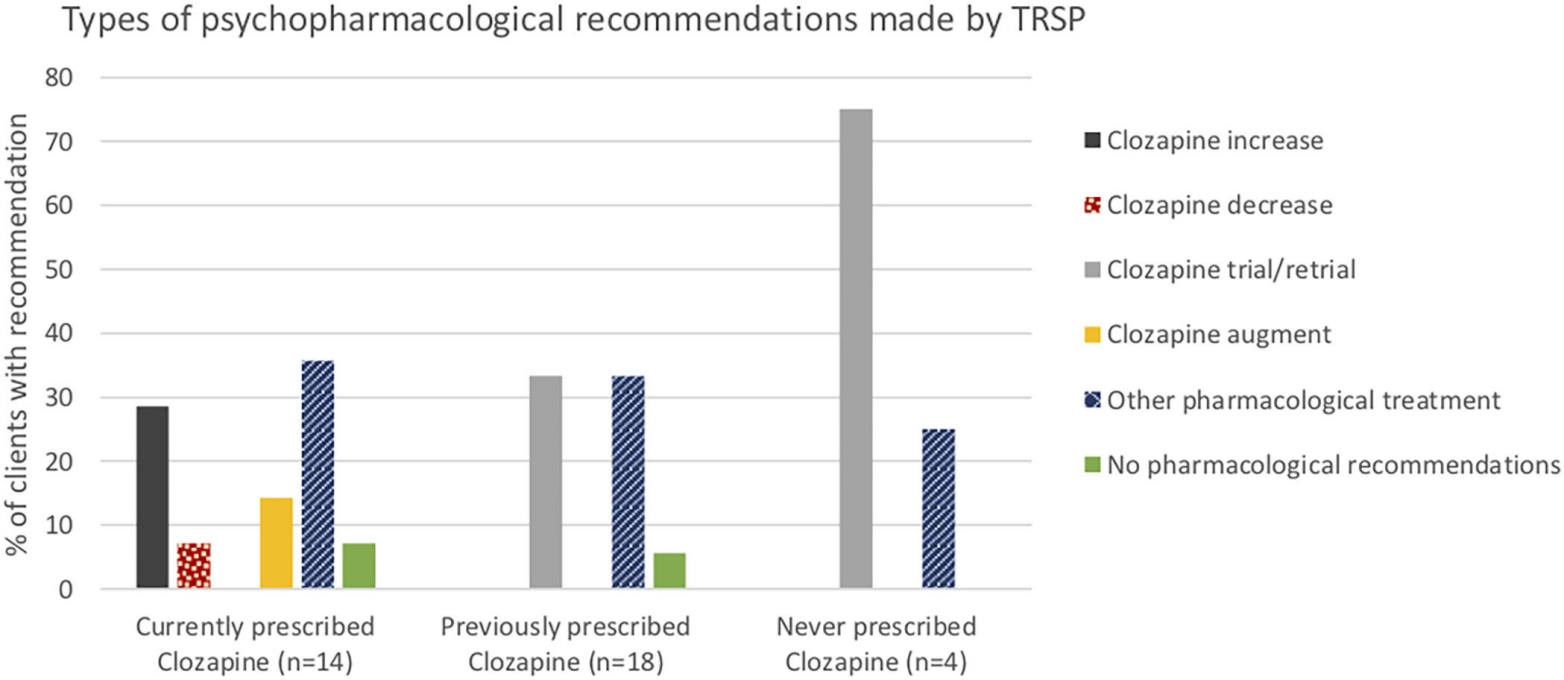
Types of psychopharmacological recommendations made by TRSP.

Psychopharmacological recommendations were made for almost all individuals (Figure 1) and varied by clozapine prescription group (Figure 2). Among individuals currently prescribed clozapine, clozapine increase was recommended in over one-quarter, and clozapine augmentation in a further 14.3%. Clozapine reduction was proposed for only one individual. Psychopharmacological treatments other than clozapine-related were recommended in over one-third (Figure 2). ECT was recommended in one (7.1%). Clozapine retrial was recommended for one-third of individuals previously prescribed clozapine, and other psychopharmacological recommendations were made in a further one-third (Figure 2). ECT was recommended in one (5.6%). In the individuals never prescribed clozapine, a clozapine trial was recommended in all but one, whose chaotic lifestyle and polysubstance use indicated, that it was likely to be unsuccessful until these lifestyle factors could be addressed (Figure 2). ECT was recommended for this individual.

Physical health and psychosocial recommendations were made for almost all individuals, and psychological interventions in one-third (Figure 1). Support for disability funding was recommended for most individuals currently prescribed clozapine, and just less than half of those previously on clozapine. Substance use interventions were recommended in a third of people currently prescribed clozapine, more commonly than in the other two groups.

## Discussion

This real-world study details the historical exposure to clozapine and ECT in individuals with treatment resistance referred to a specialist tertiary referral service for psychosis and highlights that both treatments are highly underutilized. Clozapine was currently being used in less than half of those with TR, and where it had been used previously, there was a lack of evidence that adequate clozapine trials had been delivered. There was a lack of documented evidence that plasma levels were being used to guide clinical decision-making in achieving therapeutic doses or before the use of augmentation strategies.

### Plasma monitoring

A key finding was the very limited use of monitoring plasma levels to ensure that the therapeutic threshold had been reached. Given the very high rates of individuals assessed by their clinicians to have persistent symptoms while on clozapine, it is unknown whether these individuals indeed ever received an adequate trial of clozapine. This is unfortunate because small changes to practice such as performing regular routine plasma level monitoring would enable evidence-based clinical decision-making around, e.g., an increase in dose where there has been limited clinical improvement and plasma levels are below the response threshold of 350 ng/ml (34). A threshold clozapine plasma concentration of 350–600 ng/ml has been proposed as necessary to achieve an adequate response (35, 36) though there is a notable inter-individual variation in this relationship. There is evidence, for example, that maintenance dosing to achieve plasma concentrations as low as 200 ng/ml is sufficient to prevent relapse (37), while for acutely unwell individuals, a plasma concentration of 650 ng/ml and higher is required to achieve the therapeutic effect (36, 38). To date, there is a lack of evidence on predictors of who will respond at lower or higher concentrations (38), so clinicians should monitor response and plasma levels regularly throughout clozapine titration and treatment. Where there is non-response at lower doses, the dosage should be increased until a threshold of at least 350 ng/ml has been reached (17). Enquiring about smoking status and offering smoking cessation may enable individuals to achieve a therapeutic level of clozapine without a need to increase the dose. Smoking in this group, as in populations with SMI generally (39), was very common.

### Prescribing practice: Augmentation and antipsychotic polypharmacy

Over half of the individuals currently receiving clozapine received additional treatment with some evidence base as an augmentation agent. No use, however, was being made of aripiprazole or amisulpride, the agents for which there is the strongest evidence of augmenting clozapine efficacy (20, 21). The overall effect size of augmentation is small at 0.24 (20) but is nonetheless worthwhile pursuing in those who have clozapine-resistant illnesses. Guidelines suggest that augmentation should be introduced only after treatment non-response has been confirmed by assessing both clinical profile and therapeutic blood level (17). In this study, there was, in most cases, insufficient evidence documented in clinical notes to conclude that an adequate trial of clozapine had been achieved, and thus prescription of augmentation agents should be deemed premature. Use of the cytochrome P450 (CYP) 1A2 receptor inhibitor fluvoxamine, though rare, did not appear to be complemented by routine plasma level monitoring. Concomitant use of fluvoxamine and clozapine can be hazardous, indeed potentially fatal, leading to a recommendation against their routine co-prescription (40). Where fluvoxamine and clozapine are prescribed together, good practice would be to employ regular frequent plasma monitoring to ensure therapeutic levels of clozapine do not become supra-therapeutic, given the potential of fluvoxamine to variably increase plasma levels with consequent adverse effects (27).

High rates of polypharmacy were present among individuals currently prescribed clozapine. The risks among individuals with SMI of premature mortality (41) and cardiometabolic comorbidity are well established (42, 43) as is evidence of clozapine being among the antipsychotics most likely to give rise to metabolic adverse effects (44). This sample had very high rates of physical comorbidities, increasing the risk for premature death. This common exposure to potentially hazardous polypharmacy is likely avoidable in many with more careful observation of evidence-based approaches to monitor clozapine response, which should initially involve clozapine monotherapy (2).

### Prior use of clozapine

Almost all individuals who had been prescribed clozapine in the past had ceased secondary to adverse side effects, or to poor or non-adherence. These findings align with those reported in other real-world studies, such as that conducted by the National Psychosis Service (45), a UK tertiary inpatient unit for individuals with treatment-resistant psychosis, which investigated the effectiveness of pharmacological treatment, including clozapine and clozapine-augmentation using the Operational Criteria (OPCRIT) system as a largely descriptive outcome measure. Key findings were that only one-quarter of treatment-resistant patients entering the service were treated with clozapine, in whom re-challenge (the majority of individuals had been trialed with clozapine previously, but this was ceased due to adverse effects, intolerability, or non-compliance) was possible, could be effectively augmented by amisulpride or mood stabilizers, such as valproate or lamotrigine, with aripiprazole as an alternative option where metabolic control might be a priority, and that the use of such strategies resulted in improvements across the domains of the OPCRIT also allowed for decreased use of long-acting injectables and first-generation antipsychotic medications. The large sample (*n* = 325) had a mean daily clozapine dose of 445 mg (*SD* = 196) daily (45) at the entry to the National Psychosis Service, well above the mean described in this study of 338 (*SD* = 131) mg, again suggestive that the individuals in this study were sub-optimally dosed. TRRIP guidelines recommend that where plasma level monitoring is not feasible, a daily clozapine dose of 500 mg should be achieved (2, 18).

Ensuring that there is effective management of adverse side effects, and regular monitoring for response against plasma levels are examples of good clinical practice that, if applied systematically, may support more individuals to achieve an adequate therapeutic trial of clozapine with a minimum of adverse effects. Many of the individuals in this study who had previously been prescribed clozapine had very short trials which averaged only months. Optimal therapeutic benefit takes time to establish the following titration: clinical response is unlikely to occur before at least 6 weeks of treatment, allowing both for time to respond and for the plateau of dose titration (46), and guidelines recommend a minimum duration of 3 months once therapeutic plasma levels are reached (17). In practice, titration to a therapeutic dose can take several months. In a large dose-titration study (47), the mean time for individuals with treatment resistance to respond to clozapine was 82 days, with a wide range (10–401 days). Importantly, however, an average of 60 days was required to titrate individuals to the dose at which they went on to respond. After the response threshold dose had been achieved, the average time to respond was 17 days. This underlines the importance of patience in slow titration up to the therapeutic dose. It seems that a key barrier to clozapine treatment in this subgroup occurred in the very early stages of clozapine introduction, highlighting the need to offer close monitoring and attentive support during this process, including responsive management of any adverse effects (40). Once established, there is a need to maintain support for ongoing receipt of clozapine, and to encourage adherence, as poor adherence and self-cessation were commonly observed.

Importantly, barriers to clozapine prescribing do not appear to include unawareness by clinicians of the evidence base for the effectiveness of clozapine. Rather, clinicians most commonly report a lack of prescribing experience, hesitancy about routine blood monitoring, and concern about clozapine's adverse effect profile (48). Mechanisms to improve prescribing confidence to overcome these barriers could include clear protocols on dosing and blood monitoring, adherence to evidence-based medicine principles, such as monotherapy, and access to advice from experienced clozapine prescribers (48).

### ECT

Despite evidence that ECT may be an effective alternative to clozapine in the management of TRS (28, 29, 49), there was very limited use of ECT in this cohort. Education about ECT addressing key areas of concern, such as memory loss, persisting cognitive issues, and negative portrayals in the media may improve the likelihood of consenting to ECT (50). ECT was suggested as a treatment for a number of individuals in the current cohort, including those currently on clozapine as an augmentation agent and those previously treated on clozapine who were adamant that they would not consider a retrial. While ECT is considered an effective treatment for individuals with psychosis (51), a better response to ECT has been reported where concomitant antiepileptic treatment is absent, and where previous use of ECT has resulted in a good response (52). These considerations, together with potential harms associated with ECT, including epileptic seizures and cognitive impairment, must be borne in mind when assessing the likely benefits of an ECT trial. There is significant geographic variation in the availability of ECT, and this may have contributed to the low use of ECT in this sample from NSW, in which almost all individuals who had received ECT (*n* = 8/9) resided in metro rather than rural and remote centers.

### Tertiary service treatment recommendations

In individuals currently prescribed clozapine, routine use of plasma monitoring to guide prescribing was emphasized, leading to an increase in clozapine for some, and a decrease in one individual who remained non-responsive despite having achieved therapeutic plasma levels and who was thus deemed clozapine resistant. Unsurprisingly, clozapine retrial was recommended frequently among individuals who had prior inadequate trials. Importantly, though, some individuals had had such a negative experience of clozapine previously that they refused to consider a re-trial, explaining why this recommendation was not made for more people. This finding aligns with evidence that practitioners frequently cite patient-related issues including concerns about adherence and tolerability and refusal of blood test monitoring as key barriers to clozapine initiation (53).

A small number of individuals had never been prescribed clozapine or ECT. In all of those, clozapine was recommended following assessment in the tertiary referral service, except in one individual who had polysubstance use and a chaotic lifestyle with evidence of non-adherence to previous treatments, for whom substance use support and ECT were offered as recommendations.

### Limitations

A limitation of this study is the relatively small sample size. The retrospective nature of data collection through casenote review meant that key information, including for TR determination and plasma level monitoring, relied on information clearly documented in the medical records. As with all real-world studies, a limitation is that formal tools examining adherence such as pill counts or blood levels were rarely used in the clinical setting. Thus, for TR determination, if the documented opinion of the clinical team responsible for treatment at that time was that adherence was adequate, then this was accepted as evidence of adherence in the determination of TR. It is possible that plasma monitoring was being conducted more regularly but if so, it would be expected to be detailed in the clinical record, particularly if it were being used as the basis for clinical decision-making. There was generally good information in the medical record regarding clozapine dose, duration, and adverse side effects. Unfortunately, the dosage is a poor predictor of plasma concentration with very large differences observed between individuals, contributed to by factors such as age, sex, ethnicity, and smoking status (46). This meant that there was typically an absence of sufficient evidence to conclude that an adequate clozapine trial had been achieved. A final limitation of the case note review was that there was often insufficient information about ECT trial characteristics, such as the number of treatments, and duration.

### Conclusion

Among individuals with treatment resistant psychotic illness referred to a tertiary service, clozapine was currently used in less than half. Clozapine trials in real-world practice typically terminated before an adequate trial of clozapine had been achieved. Plasma levels were rarely reported in casenotes and did not appear to be used routinely to guide clinical decision-making. ECT use was very rare. Strategies to optimize the use of clozapine therapy and ECT in clinical settings are needed to increase the therapeutic effectiveness of evidence-based therapies for treatment resistant psychosis.

## Data availability statement

The raw data supporting the conclusions of this article will be made available by the authors, without undue reservation.

## Ethics statement

Ethical review and approval was not required for the study on human participants in accordance with the Local Legislation and Institutional Requirements. Written informed consent for participation was not required for this study in accordance with the National Legislation and the Institutional Requirements.

## Author contributions

JL, KD, MO'D, and IW conceptualized the study and contributed to the data collection. JL, KD, and IW contributed to the design, undertook the statistical analysis and wrote the first draft of the manuscript. All authors contributed to and have approved the final manuscript.

## Funding

This work was supported by funding from the New South Wales Ministry of Health, the Commonwealth Community Grants Hub, and the Mindgardens Neuroscience Network.

## Conflict of interest

The authors declare that the research was conducted in the absence of any commercial or financial relationships that could be construed as a potential conflict of interest.

## Publisher's note

All claims expressed in this article are solely those of the authors and do not necessarily represent those of their affiliated organizations, or those of the publisher, the editors and the reviewers. Any product that may be evaluated in this article, or claim that may be made by its manufacturer, is not guaranteed or endorsed by the publisher.
